# Adjunctive Gaseous Ozone Improves Clinical and Microbiological Outcomes in Periodontitis: A Randomized Split-Mouth Study

**DOI:** 10.3390/dj14080488

**Published:** 2026-08-06

**Authors:** Alessia Pardo, Raffaele Brancato, Annarita Signoriello, Stefano Marcoccia, Elena Messina, Gloria Burlacchini, Caterina Signoretto, Giovanni Corrocher, Giorgio Lombardo

**Affiliations:** 1 Department of Surgery, Dentistry, Pediatrics and Gynecology (DIPSCOMI), University of Verona, 37134 Verona, Italy; alessia.pardo@univr.it (A.P.); raffaele.brancato@studenti.univr.it (R.B.); giovanni.corrocher@univr.it (G.C.); giorgio.lombardo@univr.it (G.L.); 2 Dentistry Unit, Mater Salutis Hospital, 37134 Verona, Italy; stefano.marcoccia@univr.it; 3 Diagnostic and Public Health Department, University of Verona, 37134 Verona, Italy; gloria.burlacchini@univr.it (G.B.); caterina.signoretto@univr.it (C.S.)

**Keywords:** periodontitis, ozone therapy, scaling and root planing, periodontal pathogens

## Abstract

**Background/Objectives:** To evaluate the clinical and microbiological effects of adjunctive gaseous ozone therapy combined with full-mouth ultrasonic debridement and scaling and root planing (FMUD–SRP) in patients with generalized periodontitis. **Materials and Methods:** Thirty-nine patients with stage II–IV generalized periodontitis were enrolled in a randomized split-mouth clinical trial. Quadrants were assigned to receive FMUD–SRP alone or FMUD–SRP plus adjunctive gaseous ozone therapy. Ozone (2100 ppm, 80% oxygen) was applied subgingivally immediately after instrumentation during three treatment sessions. Clinical parameters were recorded at baseline, 45, and 90 days. Subgingival plaque samples were analyzed using qualitative polymerase chain reaction to detect six periodontal pathogens. **Results:** Both treatments resulted in significant clinical improvements over time (*p* < 0.05). At 90 days, ozone-treated sites showed greater probing pocket depth reduction (−1.6 ± 0.8 mm vs. −1.2 ± 0.8 mm; *p* = 0.02) and clinical attachment level gain (−1.1 ± 1.0 mm vs. −0.6 ± 1.0 mm; *p* = 0.03) compared with control sites. No significant intergroup differences were observed for bleeding on probing, plaque index, or gingival recession. A significantly greater reduction in the detection frequency of periodontal pathogens was observed in the ozone group, including complete suppression of *Treponema denticola* and marked reductions in other anaerobic species. **Conclusions:** Adjunctive gaseous ozone therapy enhances the clinical outcomes of non-surgical periodontal treatment and exerts a selective antimicrobial effect against anaerobic periodontal pathogens.

## 1. Introduction

Periodontitis is a chronic inflammatory disease characterized by progressive destruction of the tooth-supporting tissues, leading to clinical attachment loss, pocket formation, and alveolar bone resorption [1]. The disease is initiated and sustained by a dysbiotic subgingival biofilm interacting with a susceptible host immune response [2,3]. Given its high global prevalence, periodontitis represents a significant public health burden [4].

Scaling and root planing (SRP) remains the gold standard for non-surgical periodontal therapy, aiming to mechanically disrupt and remove the pathogenic biofilm and calculus deposits [5]. Although SRP is generally effective in reducing inflammation and probing pocket depth, residual periodontal pockets and persistent pathogenic microorganisms may re-main, particularly in deeper sites, potentially compromising long-term stability.

For this reason, adjunctive therapies have been investigated to enhance the clinical and microbiological outcomes of conventional mechanical instrumentation. Among these, ozone therapy has attracted interest due to its antimicrobial, anti-inflammatory, and immunomodulatory properties [6,7]. In periodontal treatment, ozone has been proposed in different formulations, including gaseous ozone, ozonated water, and ozone-based gels. Ozonated water is mainly used for irrigation because of its antimicrobial activity and biocompatibility, while ozone-based gels may provide a more prolonged local contact with periodontal tissues. Gaseous ozone allows direct delivery into periodontal pockets and may exert a rapid antimicrobial effect, particularly against anaerobic microorganisms, through oxidative damage to bacterial cell membranes and intracellular components [8]. Additionally, experimental data suggest that ozone may influence local vascular and inflammatory responses, potentially contributing to improved tissue healing [9].

Despite these biological premises, clinical evidence regarding the adjunctive use of ozone therapy in non-surgical periodontal treatment remains inconsistent. While some randomized clinical trials have reported additional benefits in probing depth reduction and inflammatory parameters, others have failed to demonstrate significant intergroup differences [10,11,12]. Moreover, heterogeneity in ozone formulations, application protocols, concentrations, and follow-up duration complicates the interpretation of existing data.

In the present study, gaseous ozone was selected because it can be applied directly into periodontal pockets immediately after mechanical instrumentation, when the disruption of the subgingival biofilm may facilitate ozone penetration. The concentration of 2100 ppm with 80% oxygen was chosen according to the clinical settings of the ozone delivery system used in the study, in order to provide a standardized and reproducible application protocol.

Therefore, the aim of the present randomized split-mouth clinical trial was to evaluate the clinical and microbiological efficacy of adjunctive gaseous ozone therapy combined with full-mouth ultrasonic debridement and scaling and root planing (FMUD–SRP), compared with mechanical therapy alone, in patients with generalized periodontitis.

## 2. Materials and Methods

### 2.1. Study Design

This study was designed as a randomized, split-mouth clinical trial with blinded outcome assessment, conducted over a 3-month follow-up period.

The study was performed in accordance with the Declaration of Helsinki and reported following the CONSORT guidelines [13]. The protocol was approved by the Ethics Committee of the University of Verona (Prog. 698CET; PD-OZONE; Prot. No. 47526) and registered at ClinicalTrials.gov (NCT07202312). All participants provided written informed consent prior to enrolment.

### 2.2. Sample Size Calculation

The sample size calculation was based on the primary outcome, defined as the intergroup difference in probing pocket depth (PPD) reduction (variation from baseline T0 to final follow-up T4) at 90 days. Based on previous systematic reviews and randomized clinical trials evaluating adjunctive ozone therapy [14,15], a clinically relevant difference of 0.4 mm with a standard deviation of 0.6 mm was assumed. With a two-sided alpha level of 0.05 and a power of 80%, a minimum of 34 sites per group was required. To account for potential dropouts, 39 patients were enrolled.

### 2.3. Study Population

Eligible participants were adults aged 18 years or older presenting with generalized stage II–IV periodontitis grade A according to the current classification of periodontal diseases [16]. To ensure adequate evaluation of treatment outcomes, patients were required to have at least four teeth per quadrant, excluding third molars, and a minimum of eight teeth with probing pocket depth (PPD) ≥ 5 mm associated with radiographic evidence of bone loss.

Patients were excluded if they presented uncontrolled systemic diseases or conditions (e.g., diabetes and smoking) that could influence periodontal status or wound healing, had received systemic antibiotic therapy or periodontal treatment within the previous six months, or were under regular treatment with antibiotics, anti-inflammatory drugs, or anticoagulant medications. Additional exclusion criteria included contraindications to topical/subgingival ozone therapy, as defined according to the manufacturer’s safety instructions and the clinical screening protocol, including known hypersensitivity to ozone or oxygen, inability to tolerate intraoral ozone application or suction devices, severe respiratory disorders that could increase the risk associated with accidental ozone inhalation, acute oral infections requiring systemic treatment, and pregnancy. Additional exclusion criteria were severe cognitive or psychiatric disorders and an American Society of Anaesthesiologists (ASA) physical status classification of III or IV.

### 2.4. Randomization and Blinding

After analysis of all teeth, which are typically recorded with six sites each in the periodontal chart for each patient, two periodontal sites were randomly assigned as unit of clinical and microbiological analysis to either the test group (FMUD–SRP plus adjunctive gaseous ozone therapy) or the control group (FMUD-SRP alone), using a computer-generated randomization sequence. Allocation concealment was ensured using sealed opaque envelopes. The examiner responsible for clinical measurements and the statistician performing data analysis were blinded to group allocation. Due to the nature of the intervention, the operator delivering ozone therapy could not be blinded.

### 2.5. Clinical Procedures

All patients underwent full-mouth ultrasonic debridement and scaling and root planing (FMUD–SRP) using ultrasonic instruments (AIRFLOW Prophylaxis Master, EMS Dental, Nyon, Switzerland) and Gracey curettes (Gracey Curettes, LM-Dental^TM^/LM-Instruments Oy, Prainen, Finland). The active non-surgical periodontal treatment was completed over three sessions performed at one-week intervals, a protocol recently evaluated in randomized clinical research on patients with stage III/IV grade C periodontitis [17]. This protocol was selected to ensure thorough mechanical debridement of all periodontal sites, improve patient comfort and treatment tolerability, and allow standardized repetition of the adjunctive ozone application during the active treatment phase.

In the test quadrants, gaseous ozone was applied immediately after instrumentation using the Ozone DTA device, distributed by Sweden & Martina S.p.A. (Due Carrare, Padua, Italy). The device was used according to the manufacturer’s instructions and was set to deliver gaseous ozone at a concentration of 2100 ppm with 80% oxygen. Ozone was delivered subgingivally into the periodontal pocket for 20 s and the application was repeated three times per session.

Clinical periodontal parameters were recorded at baseline, 45 days, and 90 days. No additional SRP or ozone applications were performed at the 45- and 90-day follow-up visits, which were scheduled only for clinical and microbiological reassessment. These follow-up intervals were selected to evaluate early and short-term periodontal healing and to monitor the possible reorganization or recolonization of the subgingival biofilm after mechanical debridement, as previous microbiological evidence showed that subgingival recolonization may occur within the first weeks to months after scaling and root planing [18]. Avoiding additional therapeutic interventions during follow-up allowed the assessment of the initial treatment effect without confounding the clinical and microbiological outcomes.

To assure the reproducibility of the protocol and strengthen the safety assessment of the intervention, the protocol used in the study for ozone administration protected both patients and operators through the use of suction systems, sealing mechanisms, ventilation. Plus, ambient ozone levels were monitored and the procedure complied with established occupational safety recommendations.

Safey of ozone levels thresholds in workplace was guaranteed by continuous monitoring through electrochemical sensors for detection of O_3_ in parts per million (ppm): these devices are typically placed near ozone generator and dental chair, together with desktop ozone detectors which show real-time levels and visual alarms if limits are overcome.

Clinical parameters were recorded at baseline, 45 days, and 90 days using a UNC-15 periodontal probe (Hu-Friedy, Chicago, IL, USA) at six sites per tooth.

Measured variables included [19]:

- Probing pocket depth (PPD)
- Clinical attachment level (CAL)
- Bleeding on probing (BOP)
- Plaque index (Löe and Silness)
- Gingival recession (REC)

The primary outcome was the intergroup difference in PPD reduction at 90 days.

At each FMUD–SRP session, all patients received individualized oral hygiene instructions and motivation according to a tailored oral hygiene approach. Instructions were reinforced at each visit and focused on toothbrushing technique, interdental cleaning devices, and home oral hygiene maintenance.

**Figure 1:**
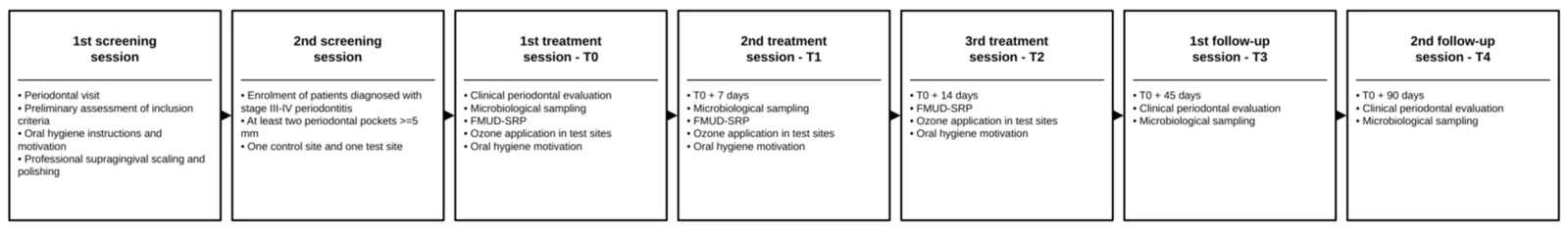
Study design and clinical workflow. Abbreviations: SRP, scaling and root planing; PPD, probing pocket depth; CAL, clinical attachment level; BOP, bleeding on probing; REC, gingival recession; PI, plaque index; T0, baseline; T1, 7 days after baseline; T2, 14 days after baseline; T3, 45 days after baseline; T4, 90 days after baseline; arrow, sequential flow of the phases.

To avoid potential bias in the clinical and microbiological outcomes, patients were instructed not to use chlorhexidine-containing mouthrinses, toothpastes, or gels, probiotics, prebiotics, or dietary supplements aimed at modulating the oral microbiota until completion of the study protocol (Figure 1).

### 2.6. Microbiological Analysis

Subgingival plaque samples were collected from periodontal pockets ≥5 mm at baseline, 45 days, and 90 days. Before sampling, supragingival plaque was carefully removed, and the selected sites were isolated with cotton rolls and gently air-dried to reduce contamination. Sterile paper points were then inserted into the periodontal pocket for 50 s and immediately transferred into sterile tubes for microbiological analysis. Samples were transported to the microbiology laboratory under controlled conditions and processed for bacterial DNA extraction. When immediate processing was not possible, samples were stored at −20 °C until analysis.

Bacterial genomic DNA was extracted using the GenElute™ Bacterial Genomic DNA Kit (Sigma-Aldrich, St. Louis, MO, USA), according to the manufacturer’s instructions. The extracted DNA was stored at −20 °C until polymerase chain reaction analysis.

Bacterial DNA was extracted and analyzed by multiplex polymerase chain reaction (mPCR), targeting species-specific sequences for detection following pathogens:

- *Aggregatibacter actinomycetemcomitans*
- *Porphyromonas gingivalis*
- *Prevotella intermedia*
- *Tannerella forsythia*
- *Treponema denticola*
- *Actinomyces naeslundii*

Microbiological outcomes were expressed as detection frequency (presence/absence) for each pathogen at each time point. As previously described [19,20], dental plaque samples were used to detect the presence of DNA from the mentioned pathogens using multiplex PCR (mPCR) (Veriti™ 96-Well Thermal Cycler Applied Biosystems, Foster City, CA, USA). Bacterial genomic DNA was extracted using the GenElute™ Bacterial Genomic DNA kit (Sigma-Aldrich, St. Louis, MO, USA) according to the manufacturer’s instructions, and the extracted DNA was stored at −20 °C until use. Two multiplex PCR (mPCR1 and mPCR2) tests were performed to detect the presence of the periodontopathogens: multiplex PCR 1, a universal 16s rRNA forward primer and a species-specific reverse primer used to detect *P. gingivalis*, *P. intermedia*, and *A. actinomycetemcomitans*; for multiplex PCR 2, specific primer pairs for each pathogen were used to detect *T. forsythia*, *T. denticola*, and *A. naeslundii*.

### 2.7. Statistical Analysis

Data were analyzed using Stata v.13.0 for Macintosh (StataCorp, College Station, TX, USA). The normality of data distribution was assessed using the Shapiro–Wilk test.

Continuous variables (PPD, CAL, REC, and PI) were expressed as mean ± standard deviation (SD). Given the normal distribution of the data, intergroup comparisons at each time point were performed using the unpaired Student’s *t*-test, while intragroup comparisons over time were assessed using the paired Student’s *t*-test.

Bleeding on probing (BOP), expressed as percentage of positive sites, was analyzed using non-parametric methods for paired data. Changes within groups were evaluated accordingly, and intergroup comparisons were performed using appropriate tests for independent proportions. For microbiological outcomes, detection frequencies (presence/absence) of periodontal pathogens were expressed as number of positive sites and percentages. Due to the lack of paired contingency data for each site, intergroup comparisons at each time point were performed using the chi-square test instead of McNemar’s test. Changes over time within groups (for Δ(T0–T4)) were evaluated using Cochran’s Q test or related non-parametric methods for repeated binary data.

The level of statistical significance was set at *p* < 0.05.

## 3. Results

Initial screening was conducted on 202 patients. At the end of the screening phase, a total of 39 patients who met the inclusion criteria were enrolled in the study. The patients presented good general health and had not undergone any periodontal treatment in the six months prior to the study. Thirty-nine patients (15 men and 24 women; mean age 53 years, range 39–74) diagnosed with generalized stage II–IV periodontitis grade A were consecutively recruited from the Department of Dentistry and Maxillofacial Surgery, University of Verona, between September 2025 and January 2026.

Among the 39 patients who agreed to participate and took part in the treatment phase:

- 2 patients did not attend the follow-up visits at T3 and T4;
- 1 patient did not attend the follow-up visit at T4.

Drop-outs thus consisted in 3 patients, 2 from T3 and 1 from T4: 78 sites (39 patients) at T0, 74 sites (37 patients) at T3 and 72 sites (36 patients) at T4 were analyzed.

Clinical and microbiological data were collected at T0 (baseline), and at the two follow-up visits at 45 days (T3) and 90 days (T4) from baseline.

### 3.1. Clinical Outcomes

**Figure 2:**
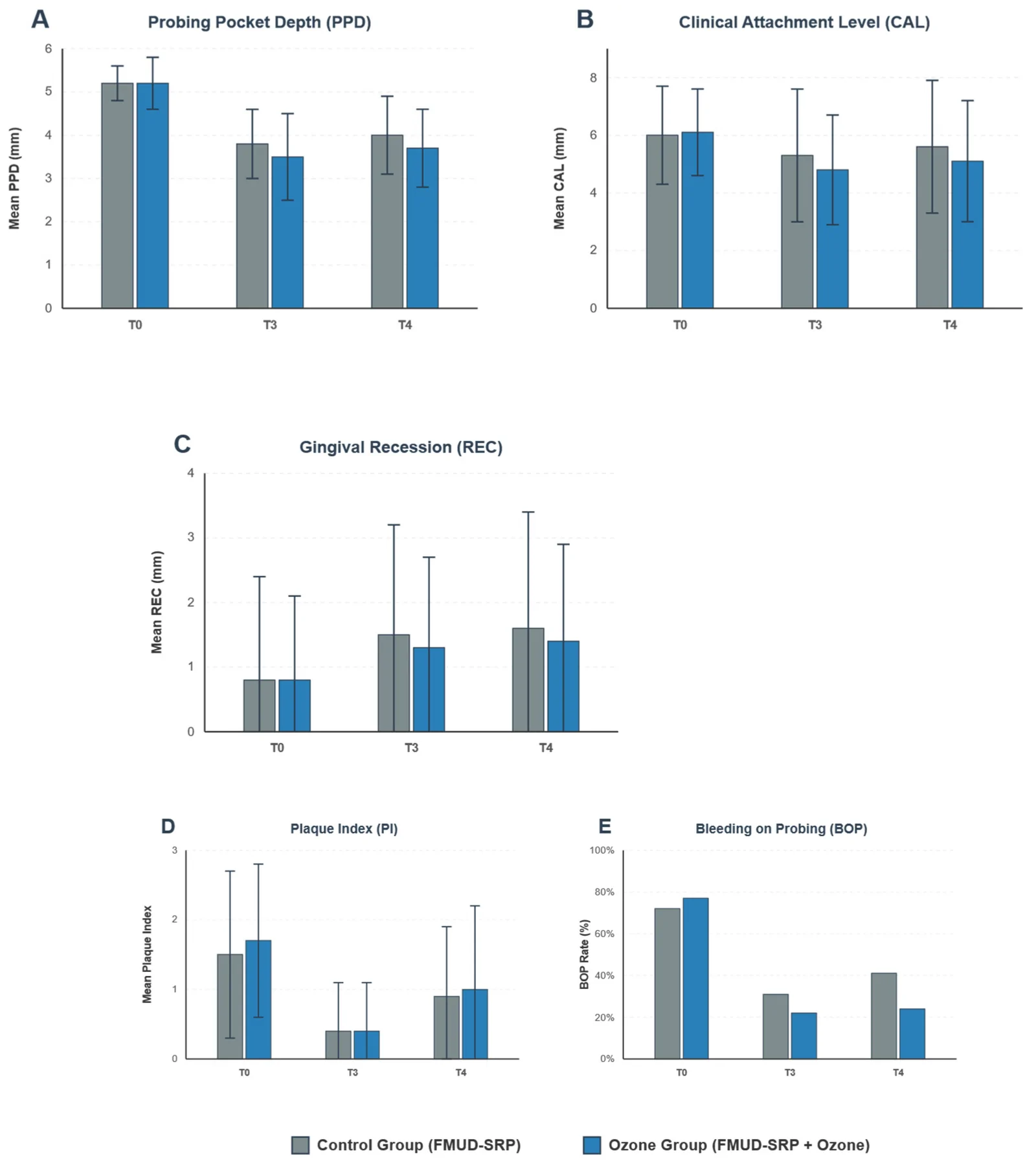
Longitudinal evaluation of clinical periodontal parameters at baseline (T0), 45 days (T3), and 90 days (T4) for the Control (FMUD-SRP) and Ozone (FMUD-SRP + Ozone) groups. (**A**) Probing Pocket Depth (PPD); (**B**) Clinical Attachment Level (CAL); (**C**) Gingival Recession (REC); (**D**) Plaque Index (PI); (**E**) Bleeding on Probing (BOP). Data in panels (**A**–**D**) are expressed as mean values with error bars representing the standard deviation (SD). Panel (**E**) shows the percentage of BOP-positive sites. * Indicates a statistically significant difference in overall changes from baseline to 90 days [Δ(T0–T4)] between the two groups (p<0.05).

All clinical parameters (PPD, CAL, REC, BOP, and PI) showed a statistically significant improvement over time in both treatment groups (*p* < 0.05), confirming the effectiveness of non-surgical periodontal therapy (Table 1 and Table 2 and Figure 2).

At baseline (T0), no statistically significant differences were observed between control and ozone-treated sites for any of the investigated clinical variables (Table 1).

A progressive reduction in probing pocket depth (PPD) was observed in both groups at 45 days (T3) and 90 days (T4). In the control group, mean PPD decreased from 5.2 ± 0.4 mm at baseline to 3.8 ± 0.8 mm at T3 and 4.0 ± 0.9 mm at T4. Similarly, in the ozone group, mean PPD decreased from 5.2 ± 0.6 mm to 3.5 ± 1.0 mm at T3 and 3.7 ± 0.9 mm at T4 (Table 1). Although no statistically significant intergroup differences were observed at individual time points, the overall reduction from baseline to 90 days [Δ(T0–T4)] was significantly greater in ozone-treated sites compared with control sites (1.5 ± 0.8 mm vs. 1.2 ± 0.8 mm; *p* = 0.02) (Table 1).

Similar trends were observed for clinical attachment level (CAL), with both groups showing a progressive improvement over time. In the control group, CAL decreased from 6.0 ± 1.7 mm at baseline to 5.3 ± 2.3 mm at T3 and 5.6 ± 2.3 mm at T4, while in the ozone group it improved from 6.1 ± 1.5 mm to 4.8 ± 1.9 mm at T3 and 5.1 ± 2.1 mm at T4 (Table 1). Although intergroup differences at single time points were not statistically significant, the overall CAL gain from baseline to 90 days was significantly greater in the ozone group (1.1 ± 1.0 mm vs. 0.6 ± 1.0 mm; *p* = 0.03) (Table 1).

With regard to gingival recession (REC), both groups showed an increase over time. In the control group, REC increased from 0.8 ± 1.6 mm at baseline to 1.5 ± 1.7 mm at T3 and 1.6 ± 1.8 mm at T4, while in the ozone group it increased from 0.8 ± 1.3 mm to 1.3 ± 1.4 mm at T3 and 1.4 ± 1.5 mm at T4 (Table 1). This increase was statistically significant within groups, likely reflecting post-inflammatory tissue shrinkage; however, no statistically significant differences were observed between groups at any time point or for overall changes from baseline (*p* > 0.05).

### 3.2. Inflammatory and Plaque Indices

Bleeding on probing (BOP) values were comparable between groups at baseline (72% in the control group and 77% in the ozone group; *p* = 0.60) (Table 2). A marked reduction in BOP was observed in both groups over time. In the control group, BOP decreased to 31% at T3 and 41% at T4, whereas in the ozone group it decreased to 22% and 24%, respectively (Table 2). Although ozone-treated sites showed a greater numerical reduction at 90 days (59% vs. 32%), the intergroup difference did not reach statistical significance (*p* = 0.06) (Figure 2E).

Plaque index (PI) values were also comparable at baseline (*p* = 0.62) (Table 2) and significantly decreased in both groups at T3 and T4 compared with baseline (*p* < 0.05). In the control group, PI decreased from 1.5 ± 1.2 at T0 to 0.4 ± 0.7 at T3 and 0.9 ± 1.0 at T4, while in the ozone group it decreased from 1.7 ± 1.1 to 0.4 ± 0.7 at T3 and 1.0 ± 1.2 at T4 (Table 2). No statistically significant intergroup differences were observed at any time point (*p* > 0.05), indicating comparable plaque control between the two treatment approaches. An overview of the longitudinal changes in all evaluated clinical periodontal parameters (PPD, CAL, REC, PI, and BOP) for both the Control and Ozone groups across the study timeline is graphically represented in Figure 2.

### 3.3. Microbiological Outcomes

At 45 days (T3) and 90 days (T4), a progressive reduction in the detection frequency of all investigated periodontal pathogens was consistently more pronounced in the ozone-treated sites (Table 3), compared to the control sites, for which an increased or unchanged frequency was observed for all cases.

At T3, ozone-treated sites already showed lower detection frequencies for all microorganisms compared with control sites, particularly for *A. actinomycetemcomitans* (7 vs. 17), *A. naeslundii* (3 vs. 14), and *T. forsythia* (6 vs. 19).

At T4, these differences became more evident. Ozone-treated sites exhibited markedly lower detection frequencies for key periodontal pathogens, including *P. gingivalis* (6 vs. 21), *A. actinomycetemcomitans* (5 vs. 13), and *T. forsythia* (7 vs. 20).

Notably, *T. denticola* was no longer detectable in ozone-treated sites at both T3 and T4, whereas it remained consistently detectable in control sites, suggesting a high susceptibility of this species to ozone therapy.

Overall, adjunctive ozone therapy resulted in a more substantial reduction in the presence of anaerobic periodontal pathogens over time.

Intergroup comparisons at 90 days revealed statistically significant differences for *P. gingivalis*, *A. naeslundii*, *T. forsythia*, and *T. denticola* (*p* < 0.05), while differences for *P. intermedia* and *A. actinomycetemcomitans* did not reach statistical significance.

## 4. Discussion

The primary objective of non-surgical periodontal therapy is to reduce the pathogenic biofilm to levels compatible with periodontal health, thereby restoring the balance between the host and the microbial ecosystem. In the present randomized split-mouth clinical trial, the adjunctive use of gaseous ozone in combination with full-mouth ultrasonic debridement and scaling and root planing (FMUD–SRP) was evaluated in comparison with mechanical therapy alone.

Overall, both treatment approaches resulted in significant improvements in all investigated clinical parameters, confirming the effectiveness of conventional non-surgical periodontal therapy, as widely reported in the literature [5]. However, adjunctive ozone therapy provided additional clinical benefits, particularly in terms of probing pocket depth (PPD) reduction and clinical attachment level (CAL) gain at 90 days. The greater reduction in PPD and improvement in CAL observed in the ozone-treated sites are consistent with previous randomized clinical trials reporting enhanced clinical outcomes when ozone is used as an adjunct to scaling and root planing [10,11]. In contrast, other studies have failed to demonstrate significant intergroup differences [12], highlighting the heterogeneity in ozone delivery systems, concentrations, and application protocols. The present findings suggest that gaseous ozone, administered subgingivally immediately after instrumentation, may exert a cumulative effect that becomes clinically evident over time, as indicated by the absence of significant differences at 45 days and the emergence of statistically significant improvements at 90 days.

Numerical increased value of CAL from T3 to T4 despite PPD decreased value can be attributed to gingival recession following resolution of inflammation and more exposed root surface. Plus, in this study the measurement variability has to be considered, as, in both groups, changes are very small (≤1 mm, as variations are of 0.3 mm). The comprehensive variation from T0 to T4 has to be considered as the most clinically relevant: there was a decrease of CAL values in both groups. In this proposal, CAL gain reflects the final and stable resolution of gingival inflammation, despite the formation of a long junctional epithelium after non-surgical therapy. In this way, CAL improvements correspond to enhanced clinical healing following periodontal debridement, due to edema reduction and firmer tissues, especially evident in ozone group.

With regard to inflammatory parameters, both groups showed substantial reductions in bleeding on probing (BOP), reflecting effective control of gingival inflammation following mechanical debridement. Although a greater numerical reduction was observed in the ozone group, the lack of statistical significance is in line with previous studies reporting variable effects of ozone on inflammatory indices [10,21]. Similarly, plaque index (PI) decreased significantly in both groups without intergroup differences, suggesting that ozone does not significantly influence supragingival plaque accumulation beyond standard mechanical therapy and patient compliance, as previously reported [22,23,24]. An increase in gingival recession (REC) was observed in both groups, which is commonly reported following successful periodontal therapy and is generally attributed to the resolution of inflammation and subsequent tissue shrinkage rather than a direct adverse effect of treatment [19]. The absence of significant differences between groups suggests that adjunctive ozone therapy does not negatively influence soft tissue healing.

At 45 days (T3) and 90 days (T4), a progressive reduction in the detection frequency of all investigated periodontal pathogens was consistently more pronounced in the ozone-treated sites (Table 3), compared to the control sites, for which an increased or unchanged frequency was observed for all cases. From a microbiological perspective, the adjunctive use of gaseous ozone was associated with a more pronounced reduction in the detection frequency of key periodontal pathogens compared with mechanical therapy alone. This finding supports the well-documented antimicrobial activity of ozone, which is primarily mediated through oxidative damage to bacterial cell membranes and intracellular components, particularly affecting anaerobic microorganisms [8]. The magnitude of the antimicrobial effect was particularly evident for *T. denticola* and *P. gingivalis*, which showed the greatest reductions in detection frequency at 90 days. Notably, *T. denticola* was no longer detectable in ozone-treated sites at both follow-up time points, suggesting a high susceptibility of this species to oxidative stress. These findings are consistent with previous microbiological studies reporting a significant reduction in anaerobic periodontal pathogens following ozone therapy [25,26,27]. The selective effect of ozone on anaerobic species may be explained by their limited antioxidant defense mechanisms, making them more vulnerable to oxidative damage compared with facultative bacteria. This selective antimicrobial action may contribute to the restoration of a more balanced subgingival microbiota, which is a key objective in the management of periodontitis [2,3], and it is also confirmed by the increased or unchanged frequency observed for control group.

Despite these promising findings, the magnitude of the observed clinical and microbiological differences should be interpreted with caution. The follow-up period was limited to 90 days, which may not be sufficient to assess the long-term stability of the treatment outcomes. Furthermore, the variability in microbiological responses reported in the literature suggests that the effectiveness of ozone therapy may be influenced by multiple factors, including treatment protocol, disease severity, and patient-related variables.

### Study Limitations

This study presents several limitations that should be considered when interpreting the findings.

First, although a priori sample size calculation was performed, the overall sample was relatively limited and may have reduced the statistical power for secondary outcomes. Larger multicentre trials would be required to confirm the magnitude of the observed intergroup differences.

Second, the follow-up period was restricted to 90 days. Given the chronic and recurrent nature of periodontitis, longer observation periods are necessary to determine the stability and sustainability of the clinical and microbiological improvements associated with adjunctive ozone therapy.

Third, ozone was administered only during the active treatment phase. The potential benefits of repeated applications during supportive periodontal therapy were not evaluated.

From a microbiological standpoint, the analysis focused on selected periodontal pathogens. However, periodontitis is currently understood as a dysbiotic biofilm-driven disease; therefore, comprehensive microbiome profiling and host-response biomarkers would provide a more complete understanding of the biological effects of ozone therapy.

In this proposal, ozone represents a broad-spectrum oxidizing agent with a non-selective antimicrobial mechanism. Although the reported reductions in *T. denticola*, *P. gingivalis* and *T. forsythia* are presented as beneficial, the microbiological analysis was limited to six pre-selected bacterial species and does not evaluate the broader effects on the subgingival microbial ecosystem. It should be acknowledged that the observed microbiological findings may represent only a partial view of the biological effects of ozone therapy and do not capture its overall ecological impact, including the potential depletion of beneficial commensal microorganisms or alterations in microbial diversity and community structure.

This issue is particularly relevant because *A. naeslundii* was included in the bacterial panel and its abundance was significantly reduced following ozone treatment (41.0% to 10.3%; *p* = 0.004). Unlike the other evaluated species, *A. naeslundii* is not consistently regarded as a periodontal pathogen; rather, it is commonly considered a commensal early colonizer associated with periodontal health and, in some contexts, protection against dental caries. Its reduction therefore raises the possibility that ozone therapy may also affect beneficial members of the oral microbiota, reinforcing the need for a more balanced interpretation of the microbiological findings and for future studies employing comprehensive microbiome analyses.

## 5. Conclusions

Within the limitations of this randomized split-mouth clinical trial, adjunctive gaseous ozone therapy resulted in significantly greater probing pocket depth reduction and clinical attachment level gain at 90 days compared with mechanical therapy alone. In addition to these clinical improvements, ozone-treated sites demonstrated a more pronounced reduction in the detection frequency of key periodontal pathogens, particularly anaerobic species, suggesting a relevant antimicrobial effect beyond conventional debridement. However, no additional benefits were observed for plaque control or bleeding on probing, indicating that the adjunctive effect of ozone may be more evident in subgingival microbial modulation rather than in supragingival or inflammatory parameters. Given the short-term follow-up and the limited sample size, these findings should be interpreted with caution. Further long-term, adequately powered clinical trials incorporating comprehensive microbiological and host-response analyses are warranted to better define the clinical relevance of ozone therapy in periodontal treatment.

## Figures and Tables

**Table 1:** Continuous variables are expressed as mean ± standard deviation (SD). Intergroup comparisons were performed using the unpaired Student’s *t*-test. Statistically significant difference (*p* < 0.05).

Parameter	Time	Control (Mean ± SD)	Ozone (Mean ± SD)	*p*-Value
PPD (mm)	T0	5.2 ± 0.4	5.2 ± 0.6	*p* = 0.74
	T3	3.8 ± 0.8	3.5 ± 1.0	*p* = 0.20
	T4	4.0 ± 0.9	3.7 ± 0.9	*p* = 0.11
	Δ(T0–T4)	1.2 ± 0.8	1.5 ± 0.8	*p* = 0.02 *
CAL (mm)	T0	6.0 ± 1.7	6.1 ± 1.5	*p* = 0.50
	T3	5.3 ± 2.3	4.8 ± 1.9	*p* = 0.45
	T4	5.6 ± 2.3	5.1 ± 2.1	*p* = 0.26
	Δ(T0–T4)	0.6 ± 1.0	1.1 ± 1.0	*p* = 0.03 *
REC (mm)	T0	0.8 ± 1.6	0.8 ± 1.3	*p* = 0.20
	T3	1.5 ± 1.7	1.3 ± 1.4	*p* = 0.80
	T4	1.6 ± 1.8	1.4 ± 1.5	*p* = 0.75
	Δ(T0–T4)	0.7 ± 0.8	0.6 ± 0.6	*p* = 0.50

**Table 2:** BOP values are expressed as percentages and were analyzed using tests for proportions. PI values are expressed as mean ± SD and were analyzed using parametric tests.

Parameter	Time	Control (Mean ± SD)	Ozone (Mean ± SD)	*p*-Value
BOP (%)	T0	72%	77%	*p* = 0.60
	T3	31%	22%	*p* = 0.42
	T4	41%	24%	*p* = 0.12
	Δ(T0–T4)	32%	59%	*p* = 0.06
PI	T0	1.5 ± 1.2	1.7 ± 1.1	*p* = 0.62
	T3	0.4 ± 0.7	0.4 ± 0.7	*p* = 0.82
	T4	0.9 ± 1.0	1.0 ± 1.2	*p* = 0.78
	Δ(T0–T4)	0.6 ± 1.6	0.7 ± 1.4	*p* = 0.97

**Table 3:** Microbiological outcomes, Δ (T4) represents the absolute difference in the number of positive sites between control and ozone groups at 90 days. Data are expressed as number of positive sites (n) and percentage (%). Intergroup comparisons at T4 were performed using the chi-square test. Statistically significant difference (*p* < 0.05).

Bacterial Species	Control T3 n (%)	Control T4 n (%)	Ozone T3 n (%)	Ozone T4 n (%)	Δ (T4)	*p*-Value (T4)
*Porphyromonas gingivalis*	17 (43.6%)	21 (53.8%)	11 (28.2%)	6 (15.4%)	15	0.0009 *
*Prevotella intermedia*	23 (59.0%)	26 (66.7%)	18 (46.2%)	18 (46.2%)	8	0.110
*Aggregatibacter actinomycetemcomitans*	17 (43.6%)	13 (33.3%)	7 (17.9%)	5 (12.8%)	8	0.060
*Actinomyces naeslundii*	14 (35.9%)	16 (41.0%)	3 (7.7%)	4 (10.3%)	12	0.004 *
*Tannerella forsythia*	19 (48.7%)	20 (51.3%)	6 (15.4%)	7 (17.9%)	13	0.004 *
*Treponema denticola*	19 (48.7%)	19 (48.7%)	0 (0%)	0 (0%)	19	<0.001 *

* = statistically significant difference between groups or time intervals.

## Data Availability

Data are available from the corresponding authors upon reasonable request.
